# Adherence to 24-h movement behavior guidelines and psychosocial functioning in young children: a longitudinal analysis

**DOI:** 10.1186/s12966-021-01185-w

**Published:** 2021-08-25

**Authors:** Rachael W. Taylor, Jillian J. Haszard, Dione Healey, Kim A. Meredith-Jones, Barry J. Taylor, Barbara C. Galland

**Affiliations:** 1 Department of Medicine, University of Otago, PO Box 56, Dunedin, New Zealand; 2 Biostatistics Centre, University of Otago, Dunedin, New Zealand; 3 Department of Psychology, University of Otago, Dunedin, New Zealand; 4 Department of Women’s and Children’s Health, University of Otago, Dunedin, New Zealand

**Keywords:** Child, Physical activity, Sleep, Screen time, 24-h movement behaviors, Mental health, Wellbeing, Psychosocial functioning

## Abstract

**Background:**

A recent paradigm shift has highlighted the importance of considering how sleep, physical activity and sedentary behaviour work together to influence health, rather than examining each behaviour individually. We aimed to determine how adherence to 24-h movement behavior guidelines from infancy to the preschool years influences mental health and self-regulation at 5 years of age.

**Methods:**

Twenty-four hour movement behaviors were measured by 7-day actigraphy (physical activity, sleep) or questionnaires (screen time) in 528 children at 1, 2, 3.5, and 5 years of age and compared to mental health (anxiety, depression), adaptive skills (resilience), self-regulation (attentional problems, hyperactivity, emotional self-control, executive functioning), and inhibitory control (Statue, Head-Toes-Knees-Shoulders task) outcomes at 5 years of age. Adjusted standardised mean differences (95% CI) were determined between those who did and did not achieve guidelines at each age.

**Results:**

Children who met physical activity guidelines at 1 year of age (38.7%) had lower depression (mean difference [MD]: -0.28; 95% CI: -0.51, -0.06) and anxiety (MD: -0.23; 95% CI: -0.47, 0.00) scores than those who did not. At the same age, sleeping for 11–14 h or having consistent wake and sleep times was associated with lower anxiety (MD: -0.34; 95% CI: -0.66, -0.02) and higher resilience (MD: 0.35; 95% CI: 0.03, 0.68) scores respectively. No significant relationships were observed at any other age or for any measure of self-regulation. Children who consistently met screen time guidelines had lower anxiety (MD: -0.43; 95% CI: -0.68, -0.18) and depression (MD: -0.36; 95% CI: -0.62, -0.09) scores at 5. However, few significant relationships were observed for adherence to all three guidelines; anxiety scores were lower (MD: -0.42; 95% CI: -0.72, -0.12) in the 20.2% who adhered at 1 year of age, and depression scores were lower (MD: -0.25; 95% CI: -0.48, -0.02) in the 36.7% who adhered at 5 years of age compared with children who did not meet all three guidelines.

**Conclusions:**

Although adherence to some individual movement guidelines at certain ages throughout early childhood was associated with improved mental health and wellbeing at 5 years of age, particularly reduced anxiety and depression scores, there was little consistency in these relationships. Future work should consider a compositional approach to 24-h time use and how it may influence mental wellbeing.

**Trial registration:**

ClinicalTrials.gov number NCT00892983

## Background

The World Health Organization defines mental health as a state of wellbeing and effective functioning in which an individual recognises his or her own abilities, is resilient to the stresses of life, and is able to make a positive contribution to his or her community [1]. Mental health difficulties can be expressed at a very early age, grouped into two broad categories of internalizing (e.g. anxiety, depression) and externalizing (e.g. hyperactivity, aggression) symptomatology. A hallmark feature of these difficulties is the ability to self-regulate, that is, exercise control over one’s thoughts, feelings, and behaviors. Self-regulation can be demonstrated in several ways, including an ability to focus and regulate attention or suppress a thought or action (inhibitory control), and in childhood, is associated with concurrent and later levels of achievement, interpersonal skills, mental health, and healthy living [2].

While many adverse environmental and social factors contribute to poor mental health in children, positive lifestyle factors such as getting enough good quality sleep, undertaking regular physical activity, and limiting time spent sedentary are also considered to be important [3–5]. However, relatively little work has examined how these behaviors influence mental health in preschool-aged children, a time of rapid growth and development. Recent reviews have highlighted the scarcity of literature (particularly that which is longitudinal in nature) and the inconsistent findings observed in the predominantly cross-sectional literature linking mental health and wellbeing outcomes with sleep [6], physical activity [7], or sedentary time [8] when each behaviour is examined in isolation.

However, a paradigm shift has occurred in the last few years which emphasises that we also need to be investigating these behaviors simultaneously because while each behavior can independently affect health, they also interact in ways that may not be apparent if studied individually [9, 10]. Consequently, many countries have introduced 24-h movement behavior guidelines for young children in recognition of this issue [11–14]. However, few studies have examined adherence to 24-h guidelines in relation to mental health in preschool-aged children [15–19], a time when sleep, physical activity and sedentary behaviors patterns are changing rapidly. The three cross-sectional studies reported some significant relationships between adherence to guidelines and improvements in behaviors, emotions [16], social cognitiion [15], or psychosocial or physical health [19]. However, in all studies, effect sizes were small and other measures evaluated showed no relationship with health outcomes [15, 16] or no effect on total health[19]. The two longitudinal studies both demonstrated no evidence of association between compliance to individual or combined guidelines and a variety of psychosocial outcomes [17, 18].

However, there are some methodological issues with these studies. While some of these studies have used objective measures of physical activity [16–18], only one assessed the impact of additional guidelines relating to activity intensity [16]. Only one of these studies also measured sleep objectively [16], although they did not examine the effect of additional guidelines relating regularity in sleep patterns. Although two studies assessed relationships between movement behaviors and mental health longitudinally [17, 18], repeated measures of movement behaviors over this time of rapid developmental change [20] have not been undertaken.

Therefore, the aim of this study was to determine how adherence to 24-h movement behavior guidelines from infancy (< 2 years) to the preschool years (2–5 years) is related to mental health and self-regulation at 5 years of age. It is hypothesized that greater time spent asleep or active, and less time spent sedentary will lead to improved mental health and a greater ability to self-regulate behaviour when children reach the age of 5 years.

## Methods

This analysis uses data from children participating in the Prevention of Overweight in Infancy (POI) randomised controlled trial undertaken in Dunedin, New Zealand. POI investigated whether anticipatory guidance for parents about infant sleep, dietary intake, and physical activity reduced excessive weight gain, compared to usual care. Anticipatory guidance is that given by the health care provider to assist parents to understand how their children grow and develop. The intervention commenced in late pregnancy and concluded when the child was two years of age [21], with follow-up measurements obtained at 3.5 and 5 years of age (just prior to starting school – children in New Zealand typically start school on or just after their fifth birthday) [22]. As the intervention did not produce differences in physical activity, sedentary behavior, or sleep [23–25], these data have been analysed using the entire cohort with appropriate adjustment for randomisation group.

As detailed information on the original trial is available in both the study protocols [21, 22] and published findings [24, 25], only brief details are provided here. The original intervention was approved by the Lower South Ethics Committee (LRS/12/08/063) and the follow-up study by the University of Otago Human Ethics Committee (12/274) and was powered to detect differences in body mass index (BMI) between intervention groups. Written informed consent was obtained from the parent/guardian of all child participants. All mothers who had booked into the single maternity hospital (> 97% of all births) serving the population of Dunedin, New Zealand were invited to participate when in mid to late pregnancy (May 2009 to December 2010). A 58% response rate yielded a final sample size of 802 primaparous (47.6%) and multiparous (52.4%) mothers. Women were randomized to one of four study groups by the study biostatistician, within 6 strata depending on area level deprivation (3 levels) and parity (2 levels), using a block size of 12. Allocation was concealed using opaque, pre-sealed envelopes. All anthropometric assessments and accelerometry analyses were performed by researchers blinded to group allocation. Demographic information obtained at baseline (late pregnancy) included maternal age, education, ethnicity, self-reported pre-pregnancy height and weight, and area level deprivation calculated using NZDep 2013 [26] (a deprivation score calculated from census data which reflects the extent of material and social deprivation, and ranges from 1 (least deprived) to 10 (most deprived)). Information on infant gestational age, sex and birth weight was obtained from hospital records. Anthropometric measurements were obtained by trained measurers following standard protocols at all subsequent time points [27]. Duplicate measures of weight (Tanita WB-100 MA/WB-110 MA) and height (Harpenden stadiometer, Holtain Ltd, UK) were obtained with children wearing underwear/light clothing [22]. Body mass index (BMI) z-scores were determined using the World Health Organization growth standards [28], with overweight defined as a BMI z-score ≥ 85^th^ but < 95^th^ percentile, and obesity as BMI ≥ 95^th^ percentile.

*Sleep and physical activity* were assessed with children wearing Actical (Mini-Mitter, Bend, OR) accelerometers 24-h a day for up to 7 days at 1, 2, 3.5 and 5 years of age. The accelerometers were worn around the waist on elastic belts and initialized using 15 s epochs. Data were cleaned and scored using an automated script developed in MATLAB (MathWorks, Natick, MA, USA) [29]. This count-scaled algorithm estimates sleep onset (start of the first 15 continuous minutes of sleep preceded by 5 min of awake) and offset (last of 15 continuous minutes of sleep followed by 5 min of awake) specific to each individual for each day. Night sleep duration is calculated as the duration of time between sleep onset and offset, excluding time awake [30]. Day-time sleep was determined at 1 and 2 years of age by the automated Matlab script which defines naps during daytime wake periods (9am to 5 pm) as at least 30 min of continuous minutes of sleep, preceded by 5 min of awake [29]. All sleep data reported here refer to total sleep duration (night-time sleep and naps, where relevant, combined). Non-wear time, sedentary time, and time in light to vigorous physical activity is then determined during awake hours only. Non-wear time was defined as at least 20 min of consecutive zeros [31]. We defined sedentary time as 0–6 counts (per 15 s), and light-to-vigorous physical activity (LMVPA) as ≥ 7 counts [32, 33]. A day was considered valid if the participant had at least eight hours of wear time outside of sleep time. Participants were only included in these analyses at each age if they had at least three valid days of data. Data from all valid days were averaged, weighted depending on whether the day was a weekend day or a weekday.

*Screen time* was assessed at 1, 2, and 5 years of age via interviewer-administered questionnaires with the parent/guardian (> 99% mothers). At 1 and 2 years of age, parents indicated the frequency per week, and the number of minutes each time, their child ‘usually’ watched television, videos or DVDs. We expanded the number of media types measured when the children were 5 years of age to include television (free to air, cable, and online streaming), DVDs or videos, computers for games (not schoolwork), passive games consoles (including handheld), and mobile phones or tablets for playing games. Parents indicated the frequency per week and duration each time their child spent watching/using each category of media use resulting in a total duration per day (minutes). Screen time information was not collected at 3.5 years of age.

*Restraint* was measured at 1 and 2 years of age only, where parents indicated the number of times over the past week, and the length of time each session, the infant was placed in a car seat or stroller.

### Adherence to guidelines

Adherence to the Canadian guidelines for the early years (0–4 years) [11] was calculated using all available data at each age, including the 5 year old measurements given most (79.9%) children were measured before their 5th birthday (mean age 4.98 (0.4) years). Level 1 adherence was defined as: *physical activity*; at least 180 min of light-to-vigorous physical activity (LMVPA) each day (average over all relevant days of measurement) at 1, 2, 3.5 and 5 years, *sedentary*; no screen time (1- and 2-year-old children) or ≤ 60 min per day (5-year-old children), and *sleep*; receiving 11–14 h (1- and 2-year-old children) or 10–13 h (3.5 and 5-year-old children) every 24 h. We also calculated Level 2 adherence guidelines as follows: *physical activity*; including at least 60 min of energetic play (defined as moderate-to-vigorous activity, MVPA) in the 3.5 and 5-year-old physical activity recommendations, *sedentary*; restricting the use of restraint (such as in car seats or strollers) for no more than an hour at a time at 1 and 2 years only, and *sleep*; having consistent bed and wake times (calculated as a within-person SD for sleep onset and offset of < 30 min) at all four ages.

### Mental health outcomes at 5 years of age

Mental wellbeing and resilience were measured by parental report using the Anxiety (13 items, covering levels of everyday worry, fear, and perfectionism α = 0.82), Depression (11 items, covering mood and negative cognitions, α = 0.77), and Resilience (12 items, covering adjustment to setbacks or unexpected events and social functioning, α = 0.82) subscales of the Behavioral Assessment System for Children (BASC-2) 2–5 year old scale [34]. The BASC-2 is a well-validated and normed scale designed to assess wide-ranging areas of child functioning as rated by parents. Self-regulation was measured using the BASC-2 Hyperactivity (11 items, covering activity level, impulsivity, and self-control, α = 0.79), Attentional Problems (6 items, covering the ability to attend and follow instructions, α = 0.80), Emotional Self-Control (6–8 items, covering the ability manage negative emotions and frustrations, α = 0.79), and Executive Functioning (10–13 items, covering inhibitory control, distractibility, and emotional reactivity, α = 0.79) subscales; as well as objective measures of Inhibitory control using the ‘Statue’ component of the NeuroPSYchological Assessment (NEPSY-2) [35] which measures difficulty with inhibitory control, and the Head-Toes-Knees-Shoulders task [36]. The NEPSY-2 is a test battery assessing numerous areas of neuropsychological functioning. It is well-normed, reliable, and appropriate for use with 5-year-old children. The Head-Toes-Knees-Shoulders task [36] is a measure of behavioral regulation and inhibition, which determines the ability of a child to follow opposing instructions (e.g. touch the head when directed to touch the toes) that is designed for 3–7 year old children.

### Statistical analyses

All statistical analysis was undertaken in Stata 16.1 (StataCorp, Texas). Only participants who had completed the 5-year assessments of mental health, had at least one assessment of movement behaviors, and had complete data on covariates (randomised group, sex, maternal education, household deprivation, primiparity, and BMI z-score) were included in these analyses. Demographics were described for the subsamples used at each age. As all mental health assessments were scored using different scales, all scores were standardised to be in units of standard deviations (SD), with the exception of the heads-toes-knees-shoulder task, which had right-skewed data that were not normalized by log-transformation and is described using the median, and the 25^th^ and 75^th^ percentiles. For ease of interpretation, three scales were reverse scored compared to the BASC-2 scoring protocol so that high scores represented more of the behavior (Resilience, Emotional self-control, and Executive functioning).

Mean differences with 95% confidence intervals (CI) in the standardised scores between those who met the guideline and those who did not meet the guideline were estimated using linear regression models, adjusted for the covariates listed previously. Quantile regression for the median was used for the heads-toes-knees-shoulder assessment. Estimates were not calculated if the group sizes were too small – this was decided to be the case if one of the groups was less than 10% of the sample. Associations are judged on the size of the mean difference as well as the 95% CI, and not solely on whether the p-value passes the threshold of ‘statistical significance’ (< 0.05) [37]. Here we use the convention that an effect size greater than 0.2SD has the potential to be meaningful [38]. Residuals of models were plotted and visually assessed for heteroskedasticity and normality.

## Results

Although 528 participants had at least one measurement of movement behaviors at any age and mental health outcomes at 5 years of age, the number of participants included at each age ranged from 256 (2 years of age) to 382 (5 years of age). The demographics at baseline across these samples were broadly comparable, with 44–51% of all infants being female and first-born. A high proportion of mothers had tertiary education, and a lower proportion of children came from homes with higher levels of deprivation than is nationally expected (Table 1). Participants included in these analyses (*n* = 528) did not differ in terms of baseline demographics from those not followed up (*n* = 274) for infant sex, maternal ethnicity, parity, or household deprivation [39]. Table 2 presents the possible minimum and maximum scores for each index, along with the mean (SD) scores for each of the mental health measures at 5 years of age.

**Table 1:** Demographics of the analysed sample at 1, 2, 3.5, and 5 years of age^a^ (*n* = 528)

		Age (years)				
		All	1	2	3.5	5
N		528	315	256	305	382
Maternal age at child’s birth, mean (SD) years		32.6 (4.6)	32.9 (4.5)	32.9 (4.4)	33.0 (4.5)	32.8 (4.5)
Primiparous, n (%)		249 (47.2)	159 (50.5)	112 (43.8)	134 (43.9)	170 (44.5)
Maternal ethnicity, n (%)	European	462 (87.5)	282 (89.5)	229 (89.5)	270 (88.5)	337 (88.2)
	Māori	21 (4.0)	11 (3.5)	6 (2.3)	11 (3.6)	14 (3.7)
	Other	45 (8.5)	22 (7.0)	21 (8.2)	24 (7.9)	31 (8.1)
Maternal tertiary education, n (%)		359 (68.0)	224 (71.1)	180 (70.3)	205 (67.2)	258 (67.5)
Household deprivation	Low	196 (37.1)	116 (36.8)	104 (40.6)	122 (40.0)	142 (37.2)
	Medium	231 (43.8)	142 (45.1)	106 (41.4)	130 (42.6)	168 (44.0)
	High	101 (19.1)	57 (18.1)	46 (18.0)	53 (17.4)	72 (18.9)
Infant sex, n (%) female		256 (48.5)	155 (49.2)	117 (45.7)	146 (47.9)	191 (50.0)
BMI z-score at 5 years of age, mean (SD)		0.46 (0.89)	0.41 (0.84)	0.43 (0.87)	0.47 (0.87)	0.48 (0.89)

^a^Analysed sample determined by those with data on mental wellbeing at 5 years of age, all demographics reported, and physical activity data at other ages

**Table 2:** Mental wellbeing, self-regulation and inhibitory control measures at 5 years of age (*n* = 528)

Outcome	Subscale	n	Possible minimum and maximum scores	Mean (SD)
Mental wellbeing and adaptive skills^a^	Depression	528	0 to 33	7.2 (3.5)
	Anxiety	528	0 to 39	8.8 (4.6)
	Resilience	528	0 to 36	25.6 (4.8)
Self-regulation^a^	Attentional problems	528	0 to 18	6.4 (2.5)
	Hyperactivity	528	0 to 33	9.5 (4.0)
	Emotional self-control	528	0 to 24	18.6 (3.1)
	Executive functioning	528	0 to 39	27.0 (4.2)
Inhibitory control^b^	Statue	463	0 to 30	17.1 (8.8)
	Head-toes-knees-shoulders	463	0 to 60	27 (7, 40)^c^

^a^Mental wellbeing, adaptive skills, and self-regulation all assessed using the Behavioral Assessment System for children (BASC-2) [34], with higher scores reflecting higher levels of construct

^b^Inhibitory control assessed using laboratory based tasks. The ‘Statue’ assessment from the NeuroPSYchological Assessment (NEPSY-2) [35] was scored from 0 (movements throughout assessment) to 30 (no movements). The Heads-toes-knees-shoulder [36] assessment was scored from 0 (all incorrect with no self-correction) to 60 (all correct)

^c^median (25th, 75th percentile) reported for Head-toes-knees-shoulders assessment because of right-skew

Table 3 presents the standardised mean difference (95% CI) in mental wellbeing scores according to whether children met each of the physical activity, sedentary time, and sleep guidelines, and combinations thereof, at each age. The 38.7% of children who participated in at least 180 min of light-to-vigorous physical activity (LMVPA) each day at 1 year of age had lower scores for depression (mean difference [MD]: -0.28; 95% CI: -0.51, -0.06) and anxiety (MD: -0.23; 95%CI: -0.47, 0.00) and higher scores for resilience (MD: 0.23; 95%CI: -0.01, 0.46) at 5 years of age than children who did not meet this guideline. Corresponding effect sizes could not be calculated at 2, 3.5 or 5 years of age because of ceiling effects in compliance (94.1 to 98.4% of children). However, fewer children met PA guidelines at these ages once the requirement to have 60 min of energetic play was included within the 180 min of LMVPA (54.1 to 81.2%), but few comparisons at this age met our requirement for a difference of at least 0.20 to be potentially meaningful, with no differences being statistically significant.

**Table 3:** Meeting 24-h movement behavior guidelines through early childhood and mental wellbeing (depression and anxiety) and adaptive skills (resilience) at five years (*n* = 528)

	n	N (%) who met guideline	Depression at 5 years of age	Anxiety at 5 years of age	Resilience at 5 years of age
			Standardised mean difference (95% CI)^a^ for those who met the guideline compared to those who did not		
Physical activity: ≥ 180 min/day LMVPA^b^					
At 1 year of age	315	122 (38.7)	**-0.28 (-0.51, -0.06)**	**-0.23 (-0.47, 0.00)**	0.23 (-0.01, 0.46)
Physical activity: ≥ 180 min/day LMVPA including 60 min energetic play^c^					
At 3.5 years of age	305	187 (61.3)	-0.04 (-0.28, 0.20)	-0.04 (-0.28, 0.20)	0.05 (-0.17, 0.28)
At 5 years of age	382	310 (81.2)	-0.11 (-0.38, 0.16)	-0.20 (-0.46, 0.06)	0.21 (-0.04, 0.47)
At both 3.5 and 5 years of age	266	144 (54.1)	-0.12 (-0.37, 0.13)	-0.15 (-0.40, 0.10)	0.17 (-0.07, 0.40)
Sedentary time: no screen time or no more than 60 min/day^d^					
At 1 year of age	305	191 (62.6)	-0.15 (-0.38, 0.08)	**-0.30 (-0.54, -0.06)**	0.12 (-0.11, 0.36)
At 2 years of age	220	35 (15.9)	-0.29 (-0.66, 0.07)	-0.17 (-0.55, 0.21)	0.13 (-0.25, 0.52)
At 5 years of age	377	151 (40.1)	**-0.34 (-0.56, -0.13)**	-0.13 (-0.34, 0.09)	0.18 (-0.03, 0.39)
At 2 or more time points	311	83 (26.7)	**-0.43 (-0.68, -0.18)**	**-0.36 (-0.62, -0.09)**	0.24 (-0.02, 0.50)
Sedentary time: not being restrained for > 60 min at a time^e^					
At 1 year of age	305	273 (89.5)	-0.14 (-0.51, 0.22)	0.00 (-0.38, 0.39)	0.07 (-0.31, 0.44)
Sedentary time: no screen time or no > 60 min AND not being restrained for > 60 min at a time					
At 1 year of age	305	176 (57.7)	-0.12 (-0.34, 0.11)	**-0.29 (-0.53, -0.06)**	0.11 (-0.12, 0.34)
Sleep: 11 – 14 h or 10 – 13 h including naps^f^					
At 1 year of age	307	258 (84.0)	-0.07 (-0.37, 0.23)	**-0.34 (-0.66, -0.02)**	0.13 (-0.18, 0.43)
At 5 years of age	370	325 (87.8)	0.18 (-0.14, 0.50)	0.00 (-0.31, 0.32)	-0.12 (-0.43, 0.19)
Sleep: consistent sleep and wake times^g^					
At 1 year of age	307	56 (18.2)	-0.20 (-0.49, 0.07)	-0.13 (-0.43, 0.17)	0.25 (-0.04, 0.54)
At 2 years of age	254	67 (26.4)	0.11 (-0.17, 0.40)	0.13 (-0.16, 0.43)	-0.06 (-0.36, 0.23)
At 3.5 years of age	398	59 (19.8)	0.13 (-0.16, 0.43)	0.15 (-0.14, 0.44)	-0.04 (-0.32, 0.24)
At 5 years of age	370	84 (22.7)	-0.21 (-0.46, 0.04)	-0.08 (-0.32, 0.17)	0.08 (-0.16, 0.33)
At 2 or more time points	380	58 (15.3)	-0.14 (-0.43, 0.14)	-0.11 (-0.40, 0.18)	0.04 (-0.24, 0.32)
Sleep: both sleep guidelines (duration and consistency)					
At 1 year of age	307	42 (13.7)	-0.25 (-0.57, 0.07)	-0.19 (-0.53, 0.16)	**0.35 (0.03, 0.68)**
At 2 years of age	254	64 (25.2)	0.11 (-0.18, 0.40)	0.10 (-0.19, 0.40)	-0.06 (-0.36, 0.24)
At 3.5 years of age	298	56 (18.8)	0.22 (-0.08, 0.51)	0.21 (-0.09, 0.51)	-0.11 (-0.40, 0.18)
At 5 years of age	370	77 (20.8)	-0.20 (-0.46, 0.05)	-0.03 (-0.29, 0.22)	0.03 (-0.22, 0.29)
At 2 or more time points	380	48 (12.6)	-0.13 (-0.44, 0.17)	0.01 (-0.30, 0.33)	-0.04 (-0.34, 0.26)
All three guidelines: ≥ 180 min/day LMVPA, no screen time or no more than 60 min, and 11 – 14 h or 10 – 13 h including naps^h^					
At 1 year of age	297	60 (20.2)	-0.19 (-0.47, 0.09)	**-0.42 (-0.72, -0.12)**	0.28 (-0.01, 0.57)
At 2 years of age	218	33 (15.1)	-0.23 (-0.60, 0.14)	-0.13 (-0.52, 0.26)	0.09 (-0.31, 0.49)
At 5 years of age	365	134 (36.7)	**-0.25 (-0.48, -0.02)**	-0.11 (-0.33, 0.11)	0.12 (-0.11, 0.34)

^a^Assessed using the Behavioral Assessment System for children (BASC-2) [34]

^a^Mean difference (95% CI) estimated using regression models adjusted for randomised group, sex, maternal education, household deprivation, primiparous, and BMI z-score at 5 years of age, in units of standard deviations. Greater scores indicate higher levels of depression, anxiety, or resilience

^b^Proportions who met the first physical activity guideline at 2, 3.5, and 5 years of age were too high to allow estimation of mean differences (94.1, 97.1, and 98.4%, respectively)

^c^Second physical activity guideline applies only to children aged 3 – 5 years

^d^No screen time (1 – 2 years) or 60 min maximum screen time (3 – 5 years). Not measured at 3.5 years

^e^Not being restrained in a car seat or stroller for > 60 min at a time, on at least 3 days a week (1 – 2 years). The proportion who met this guideline at 2 years of age was too high to allow estimation of mean differences (96.8%). Not measured at 3.5 years

^f^11 – 14 h (1 – 2 years) or 10 – 13 h (3 – 5 years). The proportion who met this guideline at 2 and 3.5 years of age and at 2 or more time points was too high to allow estimation of mean differences (92.9%, 95.6%, and 92.6% respectively)

^g^Having a consistent sleep time or consistent wake time defined as within-person standard deviation of < 30 min

^h^The proportion who met this guideline at two or more time points was too low to allow estimation of mean differences (9.7%)

Bold figures denote *P* < 0.05

Greater consistency was observed in relation to compliance with limiting screen time, in that scores for anxiety and depression were often about one-third of a standard deviation lower in children who met screen time behavior guidelines than those who did not. Effects on anxiety (MD: -0.43; 95% CI: -0.68 to -0.18) and depression (MD: -0.36; 95% CI: -0.62, -0.09) were particularly marked for those who met the screen time guidelines on at least two occasions. By contrast, restricting screen time at any single age appeared to have little effect on resilience scores, unless children had consistently limited screen use (MD: 0.24; 95% CI: -0.02, 0.50). Almost all children were not restrained in carseats or prams etc. for more than one hour at a time, with no apparent relationship with scores for mental wellbeing (Table 3).

Table 3 also indicates that while approximately one-quarter of the effect sizes for sleep compliance in relation to mental wellbeing met our threshold of potentially meaningful, only two were statistically significant. Children who slept for 11–14 h at 1 year of age had lower scores for anxiety at 5 years of age (MD: -0.34; 95% CI: -0.66, -0.02) and those who also had consistent wake and sleep times at this age had higher resilience scores at 5 years (MD: 0.35; 95% CI: 0.03, 0.68). Otherwise, meeting sleep guidelines, whether examined in terms of duration (level 1) or consistency in bed and wake times (level 2), or both, had relatively little impact on wellbeing outcomes at 5 years of age.

Children who met guidelines for all three behaviors at 1, 2, or 5 years of age had lower scores for depression when they were aged 5 years, although the difference was only statistically significant in the cross-sectional analysis at 5 years of age (MD: -0.25; 95% CI: -0.48, -0.02). Effect sizes for anxiety and resilience were more variable. The 20.2% of children who adhered to guidelines for all three behaviors at 1 year of age had lower scores for anxiety at age 5 (MD: -0.42; 95% CI: -0.72, -0.12), but meaningful effect sizes were not observed at other ages. No statistically significant differences were observed for resilience, although compliance at the age of 1 was associated with potentially better resilience at age 5 (MD: 0.28; 95% CI: -0.01, 0.57, Table 3).

Adherence to physical activity or sleep guidelines at any age had little relationship to measures of self-regulation at 5 years of age (Table 4). While a few individual results met our criteria for effect sizes that were potentially meaningful, these were not consistent over time and none were statistically significant. Meeting screen time guidelines was not associated with measures of self-regulation at any individual age. However, children who met screen time guidelines on at least two points (26.7%) had significantly lower scores for hyperactivity (MD: -0.26; 95% CI: -0.52, -0.01), and significantly higher scores for emotional self-control (MD: 0.35; 95% CI: 0.11, 0.59) and executive functioning (MD: 0.28; 95% CI: 0.03, 0.53) (Table 4). Not being restrained in carseats and strollers for more than 1 h at a time at the age of 1 year was associated with lower scores for attentional problems (MD: -0.25; 95% CI: -0.60, 0.09) and hyperactivity scores (MD: -0.37; 95% CI: -0.74, -0.00), but not emotional self-control or executive functioning at 5 years of age. Similar relationships could not be examined at two years of age because almost all (96.8%) of children met this guideline (Table 4).

**Table 4:** Meeting 24-h movement behavior guidelines through early childhood and self-regulation at five years (*n* = 528)

	n	N (%) who met guideline	Attentional problems at 5 years of age	Hyperactivity at 5 years of age	Emotional self-control at 5 years of age	Executive functioning at 5 years of age
			Standardised mean difference (95% CI)^a^ for those who met the guideline compared to those who did not			
Physical activity: ≥ 180 min/day LMVPA^b^						
At 1 year of age	315	122 (38.7)	-0.18 (-0.40, 0.04)	-0.11 (-0.35, 0.12)	0.18 (-0.03, 0.40)	0.10 (-0.12, 0.33)
Physical activity: ≥ 180 min/day LMVPA including 60 min energetic play^c^						
At 3.5 years of age	305	187 (61.3)	0.00 (-0.21, 0.20)	0.16 (-0.07, 0.39)	0.02 (-0.21, 0.24)	-0.17 (-0.39, 0.06)
At 5 years of age	382	310 (81.2)	-0.09 (-0.34, 0.15)	0.15 (-0.11, 0.41)	0.05 (-0.20, 0.31)	0.02 (-0.23, 0.27)
At both 3.5 and 5 years of age	266	144 (54.1)	0.01 (-0.21, 0.22)	0.22 (-0.01, 0.46)	0.04 (-0.19, 0.26)	-0.11 (-0.34, 0.12)
Sedentary time: no screen time or no > 60 minutes^d^						
At 1 year of age	305	191 (62.6)	-0.09 (-0.31, 0.13)	-0.15 (-0.38, 0.09)	0.12 (-0.09, 0.34)	0.07 (-0.15, 0.30)
At 2 years of age	220	35 (15.9)	-0.04 (-0.39, 0.31)	0.00 (-0.37, 0.37)	0.12 (-0.22, 0.47)	0.13 (-0.25, 0.50)
At 5 years of age	377	151 (40.1)	-0.14 (-0.34, 0.06)	-0.07 (-0.28, 0.15)	0.17 (-0.03, 0.38)	0.14 (-0.07, 0.35)
At 2 or more time points	311	83 (26.7)	-0.11 (-0.35, 0.14)	**-0.26 (-0.52, -0.01)**	**0.35 (0.11, 0.59)**	**0.28 (0.03, 0.53)**
Sedentary time: not being restrained for > 60 min at a time^e^						
At 1 year of age	305	273 (89.5)	-0.25 (-0.60, 0.09)	**-0.37 (-0.74, -0.00)**	0.14 (-0.21, 0.48)	0.19 (-0.17, 0.55)
Sedentary time: no screen time or no > 60 min AND not being restrained for > 60 min at a time						
At 1 year of age	305	176 (57.7)	-0.12 (-0.34, 0.09)	-0.15 (-0.38, 0.08)	0.10 (-0.11, 0.31)	0.05 (-0.17, 0.28)
Sleep: 11 – 14 h or 10 – 13 h including naps^f^						
At 1 year of age	307	258 (84.0)	-0.01 (-0.30, 0.28)	-0.05 (-0.36, 0.26)	0.07 (-0.21, 0.35)	0.09 (-0.21, 0.39)
At 5 years of age	370	325 (87.8)	0.14 (-0.15, 0.44)	0.04 (-0.28, 0.35)	-0.03 (-0.34, 0.28)	-0.12 (-0.42, 0.18)
Sleep: consistent sleep and wake times^g^						
At 1 year of age	307	56 (18.2)	-0.19 (-0.47, 0.08)	-0.04 (-0.33, 0.25)	0.21 (-0.06, 0.48)	0.17 (-0.11, 0.45)
At 2 years of age	254	67 (26.4)	0.09 (-0.18, 0.36)	0.01 (-0.28, 0.30)	-0.12 (-0.40, 0.15)	-0.09 (-0.38, 0.20)
At 3.5 years of age	398	59 (19.8)	-0.04 (-0.30, 0.21)	0.07 (-0.21, 0.34)	-0.14 (-0.41, 0.14)	0.02 (-0.26, 0.29)
At 5 years of age	370	84 (22.7)	-0.18 (-0.41, 0.05)	-0.19 (-0.43, 0.06)	0.16 (-0.08, 0.40)	0.13 (-0.10, 0.37)
At 2 or more time points	380	58 (15.3)	-0.19 (-0.46, 0.07)	-0.10 (-0.38, 0.18)	0.03 (-0.24, 0.30)	0.15 (-0.13, 0.43)
Sleep: both sleep guidelines (duration and consistency)						
At 1 year of age	307	42 (13.7)	-0.17 (-0.48, 0.14)	-0.07 (-0.40, 0.25)	0.22 (-0.08, 0.52)	0.23 (-0.09, 0.55)
At 2 years of age	254	64 (25.2)	0.05 (-0.23, 0.32)	-0.01 (-0.31, 0.28)	-0.08 (-0.36, 0.20)	-0.06 (-0.36, 0.23)
At 3.5 years of age	298	56 (18.8)	0.03 (-0.23, 0.29)	0.13 (-0.15, 0.41)	-0.23 (-0.51, 0.06)	-0.06 (-0.34, 0.22)
At 5 years of age	370	77 (20.8)	-0.12 (-0.36, 0.12)	-0.17 (-0.42, 0.08)	0.17 (-0.08, 0.42)	0.11 (-0.14, 0.35)
At 2 or more time points	380	48 (12.6)	-0.15 (-0.44, 0.14)	-0.07 (-0.37, 0.24)	0.00 (-0.30, 0.29)	0.15 (-0.15, 0.45)
All three guidelines: ≥ 180 min/day LMVPA, no screen time or no more than 60 min, and 11 – 14 h or 10 – 13 h including naps^h^						
At 1 year of age	297	60 (20.2)	-0.07 (-0.34, 0.20)	-0.08 (-0.37, 0.21)	0.17 (-0.09, 0.44)	0.08 (-0.20, 0.37)
At 2 years of age	218	33 (15.1)	0.05 (-0.31, 0.41)	0.08 (-0.30, 0.46)	0.02 (-0.34, 0.37)	0.01 (-0.37, 0.40)
At 5 years of age	365	134 (36.7)	-0.05 (-0.26, 0.16)	-0.02 (-0.24, 0.20)	0.12 (-0.10, 0.34)	0.07 (-0.15, 0.28)

^a^Mean difference (95% CI) estimated using regression models adjusted for randomised group, sex, maternal education, household deprivation, primiparous, and BMI z-score at 5 years of age, in units of standard deviations

^b^Proportions who met the first physical activity guideline at 2, 3.5, and 5 years of age were too high to allow estimation of mean differences (94.1, 97.1, and 98.4%, respectively)

^c^Second physical activity guideline applies only to children aged 3 – 5 years

^d^No screen time (1 – 2 years) or 60 min maximum screen time (3 – 5 years). Not measured at 3.5 years

^e^Not being restrained in a car seat or stroller for > 60 min at a time, on at least 3 days a week (1 – 2 years). The proportion who met this guideline at 2 years of age was too high to allow estimation of mean differences (96.8%). Not measured at 3.5 years

^f^11 – 14 h (1 – 2 years) or 10 – 13 h (3 – 5 years). The proportion who met this guideline at 2 and 3.5 years of age and at 2 or more time points was too high to allow estimation of mean differences (92.9, 95.6, and 92.6% respectively)

^g^Having a consistent sleep time or consistent wake time defined as within-person standard deviation of < 30 min

^h^The proportion who met this guideline at two or more time points was too low to allow estimation of mean differences (9.7%)

Bold figures denote *P* < 0.05

Relatively few significant differences were observed in levels of inhibitory control according to adherence to guidelines in preschoolers (Table 5). Higher levels of inhibitory control, as indicated by higher Statue scores, were observed for those who met physical activity guidelines at 3.5 (MD: 0.25; 95% CI: -0.01, 0.52) or both 3.5 and 5 years of age (MD: 0.24; 95% CI: 0.00, 0.49). Neither compliance with screen time guidelines or those targeting limits to time spent restrained appeared related to measures of inhibitory control. Similarly, although children who achieved sufficient sleep duration at the age of 5 showed higher levels of inhibitory control (MD: 0.36; 95% CI: 0.05, 0.68), as did those who had consistent sleep/wake times at 1 year of age (MD: 0.37; 95% CI: 0.06, 0.69), no other sleep comparisons were significant. Only 1 of 27 comparisons were significant for the Heads-toes-knees-shoulder task as a measure of behavioral regulation and inhibition, with scores being higher for children who were physically active at 3.5 years of age (median difference: 9.0; 95% CI: 1.4, 16.5). Overall, meeting all three behavior guidelines did not appear related to either of these measures of inhibitory control at any age.

**Table 5:** Meeting 24-h movement behavior guidelines through early childhood and inhibitory control at five years (*n* = 463)

	n	N (%) who met guideline	Statue at 5 years of age	Heads-toes-knees-shoulder at 5 years of age
			Standardised mean difference (95% CI)^a^ for those who met the guideline compared to those who did not	Median difference (95% CI)^b^ for those who met the guideline compared to those who did not
Physical activity: ≥ 180 min/day LMVPA^c^				
At 1 year of age	287	111 (38.7)	0.03 (-0.22, 0.28)	-2.3 (-10.0, 5.5)
Physical activity: ≥ 180 min/day LMVPA including 60 min energetic play^d^				
At 3.5 years of age	293	180 (61.4)	0.12 (-0.12, 0.35)	**9.0 (1.4, 16.5)**
At 5 years of age	372	304 (81.7)	0.25 (-0.01, 0.52)	1.4 (-7.6, 10.4)
At both 3.5 and 5 years of age	261	142 (54.4)	**0.24 (0.00, 0.49)**	7.3 (-0.9, 15.6)
Sedentary time: no screen time or no > 60 min^e^				
At 1 year of age	277	174 (62.8)	0.08 (-0.18, 0.33)	1.3 (-6.0, 8.6)
At 2 years of age	200	33 (16.5)	-0.21 (-0.59, 0.18)	4.0 (-7.6, 15.7)
At 5 years of age	367	146 (39.8)	0.15 (-0.07, 0.36)	-1.0 (-7.5, 5.6)
At 2 or more time points	296	80 (27.0)	0.02 (-0.24, 0.28)	5.3 (-2.4, 13.1)
Sedentary time: not being restrained for > 60 min at a time^f^				
At 1 year of age	277	246 (88.8)	0.06 (-0.33, 0.46)	5.7 (-5.5, 16.9)
Sedentary time: no screen time or no > 60 min AND not being restrained for > 60 min at a time				
At 1 year of age	277	159 (57.4)	0.08 (-0.17, 0.33)	2.5 (-5.0, 10.0)
Sleep: 11 – 14 h or 10 – 13 h including naps^g^				
At 1 year of age	280	235 (83.9)	-0.06 (-0.40, 0.27)	-5.9 (-15.7, 3.9)
At 5 years of age	360	316 (87.8)	**0.36 (0.05, 0.68)**	9.5 (-1.1, 20.2)
Sleep: consistent sleep and wake times^h^				
At 1 year of age	280	51 (18.2)	**0.37 (0.06, 0.69)**	7.3 (-1.9, 16.6)
At 2 years of age	233	60 (25.7)	0.03 (-0.28, 0.33)	-1.4 (-10.3, 7.5)
At 3.5 years of age	286	58 (20.3)	0.16 (-0.13, 0.45)	6.6 (-3.0, 16.2)
At 5 years of age	360	82 (22.8)	0.18 (-0.06, 0.43)	5.4 (-3.0, 13.9)
At 2 or more time points	361	57 (15.8)	0.12 (-0.17, 0.41)	4.5 (-4.5, 13.6)
Sleep: both sleep guidelines (duration and consistency)				
At 1 year of age	280	38 (13.6)	0.30 (-0.05, 0.66)	4.6 (-5.5, 14.8)
At 2 years of age	233	57 (24.5)	0.02 (-0.29, 0.33)	-1.0 (-9.9, 7.9)
At 3.5 years of age	286	55 (19.2)	0.19 (-0.10, 0.49)	5.6 (-4.1, 15.3)
At 5 years of age	360	75 (20.8)	0.19 (-0.07, 0.45)	2.9 (-5.8, 11.6)
At 2 or more time points	361	47 (13.0)	0.08 (-0.23, 0.40)	-0.2 (-9.5, 9.2)
All three guidelines: ≥ 180 min/day LMVPA, no screen time or no > 60 min, and 11 – 14 h or 10 – 13 h including naps^i^				
At 1 year of age	270	53 (19.6)	-0.10 (-0.42, 0.22)	1.4 (-8.0, 10.7)
At 2 years of age	198	31 (15.7)	-0.19 (-0.59, 0.21)	5.8 (-6.1, 17.7)
At 5 years of age	355	129 (36.3)	0.14 (-0.09, 0.36)	1.9 (-5.5, 9.2)

^a^Mean difference (95% CI) estimated using linear regression models adjusted for randomised group, sex, maternal education, household deprivation, primiparous, and BMI z-score at 5 years of age, in units of standard deviations

^b^Median difference (95% CI) estimated using quantile regression models adjusted for randomised group sex, maternal education, household deprivation, primiparous, and BMI z-score at 5 years of age, scores range from 0 (less inhibitory control) to 60 (more inhibitory control)

^c^Proportions who met the first physical activity guideline at 2, 3.5, and 5 years of age were too high to allow estimation of mean differences (93.6, 96.9, and 98.4%, respectively)

^d^Second physical activity guideline applies only to children aged 3 – 5 years

^e^No screen time (1 – 2 years) or 60 min maximum screen time (3 – 5 years). Not measured at 3.5 years

^f^Not being restrained in a car seat or stroller for > 60 min at a time, on at least 3 days a week (1 – 2 years). The proportion who met this guideline at 2 years of age was too high to allow estimation of mean differences (98.0%). Not measured at 3.5 years

^g^11 – 14 h (1 – 2 years) or 10 – 13 h (3 – 5 years). The proportion who met this guideline at 2 and 3.5 years of age and at 2 or more time points was too high to allow estimation of mean differences (92.3, 95.5, and 92.5% respectively)

^h^Having a consistent sleep time or consistent wake time defined as within-person standard deviation of < 30 min

^i^The proportion who met this guideline at two or more time points was too low to allow estimation of mean differences (9.8%)

Bold figures denote *P* < 0.05

## Discussion

Our longitudinal study demonstrates that achieving appropriate levels of sleep and physical activity while limiting sedentary time appears to have relatively little impact on measures of mental health and wellbeing at five years of age. Overall, meeting individual physical activity or sleep guidelines did not appear consistently related to levels of anxiety or depression in preschool-aged children, nor did this predict improved self-regulation or inhibitory control at this age. By contrast, scores for anxiety and depression were often about one-third of a standard deviation lower in children who met sedentary behavior guidelines compared with those who did not at any age. Children who met guidelines for all three behaviors tended to have lower scores for anxiety at 5 years of age, whereas findings for depression and resilience were more variable. Meeting all guidelines was not related to significant differences in self-regulation or inhibitory control at any age examined.

It is difficult to compare our findings with the literature given so few studies have examined this question in early life. Comparisons are also limited by the wide range in outcomes that have been investigated, with little consistency across the relevant studies. A recent review in older children and adolescents has suggested favourable associations between meeting all three recommendations and improved mental health when compared with meeting none of the recommendations [40]. While this appears somewhat in contrast to the current study, these authors highlight that the evidence in general is weak, and in relation to each specific mental health indicator, was limited [40]. Data in younger children are scarce. A cross-sectional study in 4-year old children reported that adherence to all three guidelines was marginally associated with better Theory of Mind performance, a measure of a child’s ability to attribute core mental states, but had no effect on emotional understanding as measured using the Test of Emotion Comprehension [15]. Another cross-sectional study in 3-year old children determined that while children meeting more guidelines did have lower scores for emotional and behavioral problems as measured using the Child Behavior Checklist, the observed effect size was small [16]. Only two longitudinal studies appear to have been undertaken, both demonstrating no evidence of association between compliance to individual or combined guidelines and a variety of psychosocial outcomes one [18] to three [17] years later. These findings fit with the mixed body of literature examining each individual behavior in relation to outcomes of interest. Although previous reviews have demonstrated that children with sleep problems are more likely to develop emotional and behavioral problems such as depression and anxiety [3, 41, 42], compliance analyses such as in the current study focus on sleep duration (and in our case also sleep regularity in terms of sleep and wake timings) rather than examining the presence or otherwise of sleep problems. In our study, scores for anxiety were lower in those who met guidelines for sleep duration at one year of age (11–14 h a night), scores for resilience were higher for those with consistent wake schedules at one year of age, and children who met both types of sleep guideline at 5 years of age showed improvements in inhibitory control. However, significant differences were not observed at any other ages or measures examined, illustrating limited consistency in the relationships observed. Others have also reported no relationship between meeting sleep guidelines and inhibition [18] or measures of externalizing and internalizing problems [16, 18] or emotional skills or prosocial behaviors scores [17], although Cliff et al. [15] reported significantly greater emotional comprehension in children who met sleep duration guidelines.

Similarly, while previous reviews have demonstrated associations between physical activity and mental health in children, the effect sizes are often very small, the research designs are often weak, and considerable heterogeneity exists [4, 43]. For example, none of the these previous 24-h movement guidelines papers found any significant relationship between meeting activity guidelines and any measurement of mental or psychosocial health examined [15–18]. A recent review in older children also demonstrated that relationships between physical activity and mental health outcomes were less apparent than those observed for sleep and screen time compliance, although the quality of the evidence was very low, with all studies examined being cross-sectional in design [40].

By contrast, our findings regarding the benefits of restricting screen time on reducing levels of depression and anxiety just prior to starting school did appear more consistent, with potentially meaningful effect sizes observed at many of the time points examined. Beneficial effects were particularly evident in children who met guidelines at two or more time points. None of the previous 24-h movement papers specifically examined anxiety or depression, with most reporting no significant relationships with a variety of other measures [15, 17, 18], with the exception of Carson et al. [16] who reported higher externalizing and internalizing problem scores in children who did not meet screen time guidelines. Previous reviews have also indicated that the relationships between screen time and psychosocial outcomes are inconsistent in young children, with few measuring anxiety or depression for comparison [44]. However, a recent review of reviews has highlighted that there is moderately strong evidence for an adverse association between screen time and depressive symptoms (with a weak association for anxiety) in children and adolescents in general [45]. Why our study observed more consistent findings for screen time compared with physical activity or sleep is not clear, particularly given the parent-reported nature of our screen time data in comparison with data on sleep and physical activity which were objectively measured. Unfortunately, little is currently known regarding the underlying mechanisms explaining how and why sleep and activity may be beneficial, and screen time not, for mental health in children [4]. Few studies have examined potential mechanisms and a high level of study heterogeneity exists, limiting firm conclusions.

It is clear that many questions remain about the relationships between sleep, physical activity, and screen time in relation to mental health and wellbeing, with a strong need for prospective, high-quality studies that use robust measures of all movement behaviors and validated measures of mental health to increase our understanding in this topic area [40]. Repeated measures of key behaviors may help address issues relating to the heterogeneity of findings at this young age, given the rapid changes in development that occur both behaviourally and emotionally. Psychosocial outcomes need to be examined in a multitude of ways, including both subjective and objective tests where possible, given the relative strengths and weaknesses of each. It is also feasible that the mixed findings to date arise as a result of insufficient consideration of whether any critical windows exist for 24-h movement behaviors being able to influence later mental health and wellbeing in young children.

Our study has several strengths including the assessment of both sleep and physical activity by actigraphy, over several days, and at multiple time points, and our evaluation of the second ‘level’ of sleep (consistency) and sedentary (restraint) guidelines which have rarely been examined to date. We also evaluated multiple aspects of mental health and wellbeing, using both parental report assessing ‘usual’ behavior, and a more ‘moment-in-time’ lab-based assessment by trained measurers. Measuring children’s self-regulation skills via laboratory tasks represents a significant strength of this study. However, our study also has some limitations. While our measurement of screen time covered multiple types of device use, reflecting the rapid changes in technology use over recent times, we did not collect quantifiable data at 3.5 years of age limiting any comparison against guidelines at this time. Although our sample size was reasonably large, we did not have sufficient participant numbers to investigate whether differences in the relationship between adherence to guidelines and mental health and wellbeing outcomes were apparent in boys and girls. We also could not assess the relationships at several of the time points because of ceiling effects with very high numbers of children achieving the requisite guideline (physical activity at 2 years, sleep at 2 and 3.5 years). It is perhaps surprising that such high numbers of children achieved the sleep guidelines given that we measured sleep using actigraphy, which typically provides values for sleep duration that are shorter than those obtained by questionnaire [46]. However, current Canadian sleep guidelines have been predominantly developed on the basis of questionnaire data on sleep duration [11], meaning that it is likely we have underestimated the number of children who met sleep guidelines. In the absence of alternatives, we used accelerometer cut-points that were developed for older toddlers [32, 33] than the youngest age point included in our sample (3 years versus 1 year) and the same cut points at each age point. As children’s motor development changes rapidly during the first two years of life, the use of these cut-points may have introduced measurement inaccuracy. Finally, multiple measures of mental health and psychosocial functioning were presented, but these were mostly obtained from a single questionnaire, the BASC-2, in which some items are used in a number of different subscales. However, while the different constructs represented by the subscales might be correlated, the subscales were examined in separate models.

## Conclusions

Although adherence to some individual movement guidelines at certain ages throughout early childhood was associated with improved mental health and wellbeing at 5 years of age, particularly reduced anxiety and depression scores, there was little consistency in these relationships. Therefore, it is not possible to conclude that better mental health and wellbeing outcomes result for pre-schoolers meeting any one, or all movement guidelines from this research. Perhaps a different analytic approach is required such as examining compositional time use [47, 48] in relation to mental wellbeing in children. Compositional analyses, a relatively recent phenomenon, investigate all movement behaviours (sleep, activity, sedentary time) simultaneously, by accounting for the finite nature of the 24-h window, where if time spent in one component increases (e.g. sleep), then another component within the same 24-h block *must* decrease (e.g. sedentary time or physical activity). While researchers are starting to examine compositional time use in relation to mental wellbeing in children cross-sectionally [49, 50], longitudinal data are scarce [51].

## Data Availability

The datasets used and/or analysed during the current study are available from the corresponding author on reasonable request.

## Abbreviations

LMVPA: Light-to-vigorous physical activity
MVPA: Moderate-to-vigorous physical activity
BASC-2: Behavioral Assessment System for Children
NEPSY-2: NEuroPSYchological Assessment
